# Determination of the parameters of rock viscoelastic creep model and analysis of parameter degradation

**DOI:** 10.1038/s41598-023-32565-w

**Published:** 2023-04-07

**Authors:** Zhiming Zheng, Yu Yang, Cheng Pan

**Affiliations:** 1 School of Civil Engineering, Liaoning Technical University, Fuxin, 123000 China; 2 School of Civil Engineering, Liaoning University of Technology, Jinzhou, 123000 China

**Keywords:** Civil engineering, Engineering

## Abstract

Different stress creep tests are conducted on the sandstone in this study to better describe the creep properties of rocks under different stress states. A model that describes the rock creep process is established. The various stages of creep can be described by combining the creep properties of the creep elements of the model. A new method for determining creep parameters is proposed by using the special point on the creep curve and the definition of creep deformation. The relationship between the creep parameters, stress, and time is analyzed. An improved creep model that considers the effects of stress state and time on the creep parameters is developed. This model is verified using experimental data and calculation results. Results shows that the improved creep model better describes the creep properties of rocks and provides a new method for determining future model parameters. The shear modulus of elastic model controls the instantaneous deformation. The shear modulus of viscoelastic model governs the limit of viscoelastic deformation. The shear viscoelastic coefficient of viscoelastic model increases with the increase in stress. The coefficient of viscoplastic model controls the viscoplastic creep rate. The coefficient of a nonlinear Newtonian dashpot mainly controls the accelerated creep deformation of rock. The calculation results of the proposed model agree well with the experimental data under the action of different stress levels. This model accurately reflects the creep characteristics of the primary and steady-state creep stages, and overcomes the shortcomings of the traditional Nishihara model in describing accelerated creep.

## Introduction

The engineering rock mass exhibits short-term elastoplastic mechanical properties under geological and human disturbance and displays time-effect-related deformation and failure properties under certain stress and strain conditions^1,2^. This time-dependent property of rock is referred to as the rheological behavior of the rock mass^3,4^. Under the influence of geological actions, natural environment change, and engineering disturbance, the rock mass gradually deteriorates over time^5–7^. This condition leads to the creation of new cracks and the expansion and development of existing cracks until they penetrate the rock. The deformation gradually increases until the rock mass loses its bearing capacity, ultimately resulting in the overall instability of the engineering rock mass^8,9^. Microscopically, the internal mineral structure of the rock mass changes and reorganizes over time under the coupling of external loads, leading to a weakening of its physical and mechanical properties^10–12^. The deformation of rock initially undergoes large and dynamic changes, then transforms into a quasi-static process of small deformation^13,14^. With the development of time and external loads, the deformation of the rock becomes a dynamic process of large deformation^15,16^. The rock exhibits a variety of macroscopic and localized phenomena.

The deterioration law of creep model parameters with time and stress was mainly obtained by fitting the established model with experimental data. The law of the change in parameters was then analyzed. Liu et al.^17^ developed a new model that describes accelerated creep in rock by replacing the viscous elements in the traditional Nishihara model with fractional elements that consider the changes in time and stress. The validity of the model was verified by fitting it to experimental data. Wang et al.^18^ studied the unloading creep characteristics of layered rock samples from Jinping II Hydropower Station and obtained the creep deformation curves under different confining pressures. They established a constitutive equation for a nonlinear creep model under 3D stress and identified the creep parameters using an optimization algorithm. The creep parameters showed a nonlinear decline with the decrease in confining pressure. Singh et al.^19^ proposed a combined creep model based on the Maxwell and Hooke models that was established using acoustic emission technology and calculated the elastic and viscous parameters of the model. The model can accurately predict the stress–strain response of rock salt under loading and unloading conditions. Hou et al.^20^ presented a new nonlinear nonlinear creep damage model that considers the influence of initial damage on the creep properties of rock. The parameters of this model were obtained through fitting method, and the creep characteristics of sandstone under different initial damage conditions were verified. Wang et al.^21^ described sandstone creep test results using a nonlinear and nonstationary plastic viscosity (NNPV) creep model. The creep parameters of the rock at three osmotic pressure levels were determined, and the theoretical curves using the NNPV model agreed well with the experimental data. However, the fitting of the creep parameters by fitting did not have obvious physical meaning.

This study presented a model that describes the rock creep process. The creep properties of each component in the model corresponded to the various stages of the classic creep curve. On the basis of the above research on the rheological properties of rock and considering the influence of stress level and time on the deterioration of creep parameters, a method for determining creep parameters was proposed by combining the deformation characteristics of creep curves. A creep model that considers the effects of stress and time on the creep parameters was established. A 1D creep model was developed by using the proposed method to promote the 3D state. The relationship between the creep parameters, stress, and time in 3D state was analyzed. The equations for each creep parameter, stress, and time were substituted into the creep model, and the model curve was compared with the test curve. The validity of the model and the rationality of the new method for determining the creep parameters were verified by calculating the high similarity between the curve and the variation of the test curve. The creep parameter model was calculated to clarify the physical meaning of the creep parameters under different stresses and different times.

## Parameter determination method for one-dimensional creep model

### Creep process characteristics and creep model establishment

**Figure 1:**
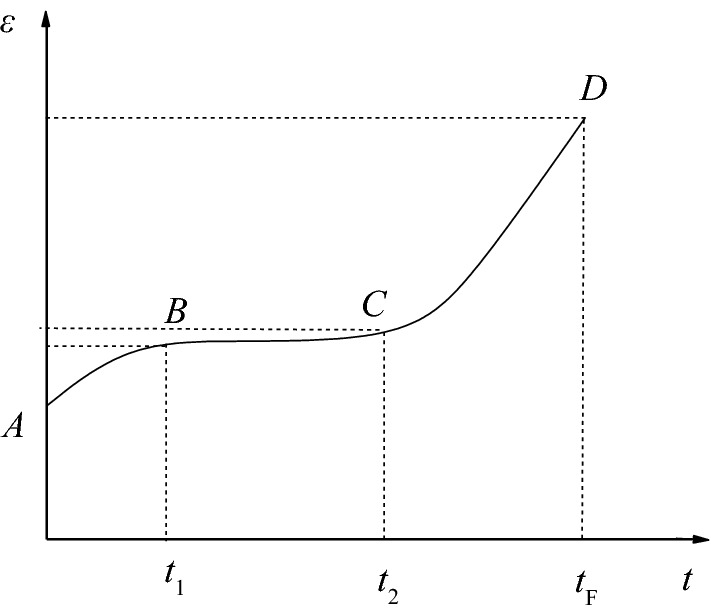
Typical creep curve.

The creep property indicates that a rock gradually deteriorates over time due to constant external force. A large number of experimental studies have shown that the creep of rock has obvious stages. The rock creep process can be divided into three stages: primary creep, steady-state creep, and accelerated creep. The schematic of the rock creep process is shown in Fig. 1^22^.

The instantaneous deformation in the 1D state can be expressed as

1 $$\varepsilon_{e} = \frac{\sigma }{{E_{1} }},$$

where *E*_1_ is the modulus of elasticity, *ε*_*e*_ is the instantaneous strain value, and *σ* is the applied creep stress.

The variation law of primary creep deformation can be described by Kelvin.

2 $$\varepsilon_{K} = \frac{\sigma }{{E_{2} }}\left[ {1 - \exp \left( { - \frac{{E_{2} }}{{\eta_{1} }}t} \right)} \right],$$

where *E*_2_ is the viscoelastic modulus, *η*_1_ is the viscous coefficient, and *t* is the creep time.

The variation law of steady-state creep deformation can be described by using a viscoplastic model^23^.

When *σ* < *σ*_*s*_,

3 $$\varepsilon_{N} = 0,$$

when *σ* ≥ *σ*_*s*_,

4 $$\varepsilon_{N} = \frac{{\sigma - \sigma_{s} }}{{\eta_{2} }}t,$$

where *η*_2_ is the viscous coefficient of the viscoplastic model, and *σ*_s_ is the long-term strength of the rock.

**Figure 2:**
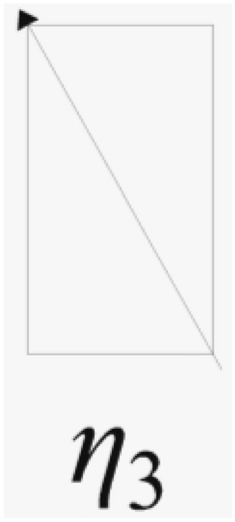
NonlinearNewtonian dashpot.

The whole process of creep deformation is the process of progressive fracture and damage accumulation in rock until crack propagation and failure. Therefore, a parameter can be introduced to characterize the irreversible deformation of rock materials. Considering the nonlinear characteristics of rock, its stress–strain response often loses one-to-one correspondence. The deformation of rock is irreversible. The strain parameter can be selected to characterize whether the rock enters the accelerated creep stage. A nonlinear Newtonian pot is introduced to describe the deformation of rock in the accelerated creep stage. Therefore,The variation of the accelerated creep deformation can be described by using an nonlinear Newtonian dashpot as shown in Fig. 2.

The model can better reflect the accelerated creep characteristics of rock, and its creep constitutive equation is

5 $$\varepsilon_{3} = \frac{\sigma }{{2\eta_{3} }}\left( {t - t_{2} } \right)^{2} ,$$

where *t*_2_ is the time to enter the accelerated creep, which can be directly obtained through experiments, and *η*_3_ is the viscosity coefficient of the nonlinear Newtonian dashpot. The unit of viscosity coefficient of the nonlinear Newtonian dashpot *η*_3_ is GPa·h^2^.

The creep deformation constitutive equation of rock in 1D state is

6 $$\left\{ {\begin{array}{*{20}c} \begin{gathered} \varepsilon = \frac{\sigma }{{E_{1} }} + \frac{\sigma }{{E_{2} }}\left[ {1 - \exp \left( { - \frac{{E_{2} }}{{\eta_{1} }}t} \right)} \right],\,\sigma < \sigma_{s} \hfill \\ \varepsilon = \frac{\sigma }{{E_{1} }} + \frac{\sigma }{{E_{2} }}\left[ {1 - \exp \left( { - \frac{{E_{2} }}{{\eta_{1} }}t} \right)} \right] + \frac{{\sigma - \sigma_{s} }}{{\eta_{2} }}t,\,\sigma \ge \sigma_{s} ,t < t_{2} \hfill \\ \end{gathered} \\ {\varepsilon = \frac{\sigma }{{E_{1} }} + \frac{\sigma }{{E_{2} }}\left[ {1 - \exp \left( { - \frac{{E_{2} }}{{\eta_{1} }}t} \right)} \right] + \frac{{\sigma - \sigma_{s} }}{{\eta_{2} }}t + \frac{\sigma }{{2\eta_{3} }}\left( {t - t_{2} } \right)^{2} ,\,\sigma \ge \sigma_{s} ,t \ge t_{2} } \\ \end{array} } \right..$$

### Creep model and division of creep stage

**Figure 3:**
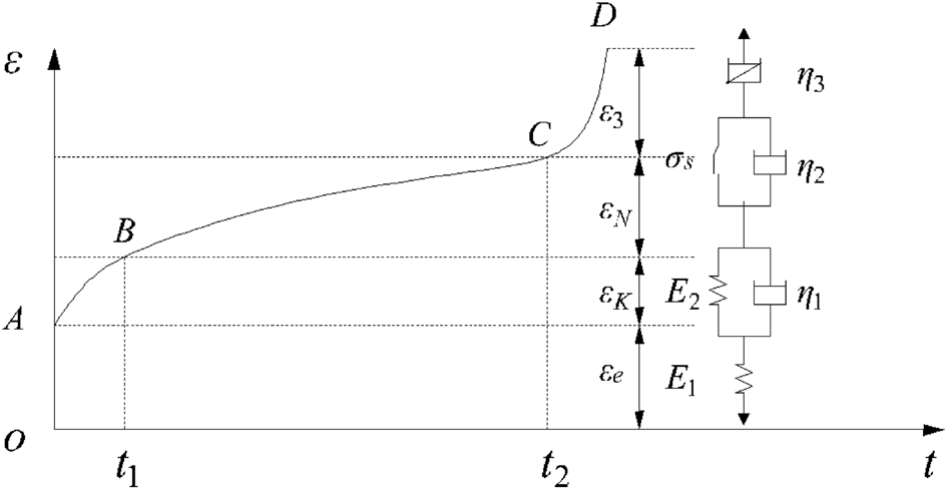
Corresponds to the model of the creep curve.

A schematic corresponding to the model and the creep curve is drawn to more vividly represent the correspondence between the components of the creep model and the deformation of each stage of the creep, as shown in Fig. 3^24,25^.

### Determination of elastic modulus E_1_

As shown in Fig. 3, the rock has a certain instantaneous strain *ε*_*e*_ at the initial moment of the creep curve under the action of stress. The instantaneous strain of the rock includes elastic and plastic strains. This part of the instantaneous strain is assumed to be elastic strain, and the elastic modulus *E*_1_ of the rock can be determined by using Eq. (1)^26^.

7 $$E_{1} = \frac{\sigma }{{\varepsilon_{e} }}.$$

### Determination of the viscosity coefficient η_2_ of viscoplastic model

Before the accelerated creep stage, rock can be called linear rheological process. Its deformation is mainly viscoelastic deformation. After rock unloading, the strain can be basically recovered. At this time, the permanent deformation is small or negligible. The accelerated creep stage can be called a nonlinear rheological process. After the accelerated creep begins, the viscoelastic deformation is stable. The permanent deformation is increasing, accounting for most of the total deformation. Therefore, rock creep is regarded as the superposition of linear creep and nonlinear creep. When the rock enters the steady-state creep stage, its primary creep strain reaches a steady state. With the progress of the creep time, the creep strain of the rock in the steady-state creep stage is the sum of the stable creep primary value and the steady-state creep strain value. The Kelvin model can better reflect the viscoelastic creep properties of the rock. Specifically, Eq. (2) can show the variation law of steady-state creep curve. At the same time, when the rock enters the accelerated creep stage, the viscoelastic deformation is basically stable. The strain increasing with time is only permanent deformation^27^. When creep time *t* tended to *∞*, the creep stability value of rock primary is obtained.

8 $$\varepsilon_{K,t \to \infty } = \mathop {\lim }\limits_{t \leftarrow \infty } \left\{ {\frac{\sigma }{{E_{2} }}\left[ {1 - \exp \left( { - \frac{{E_{2} }}{{\eta_{1} }}t} \right)} \right]} \right\} = \frac{\sigma }{{E_{2\infty } }},$$

where *E*_2∞_ is the viscoelastic modulus when the time is *∞*.

The creep deformation values of *t*_*i*_ and *t*_*j*_ at the time point are obtained by randomly taking two time points *t*_*i*_ and *t*_*j*_ on the steady-state creep curve.

9 $$\left\{ \begin{gathered} \varepsilon_{i} = \frac{\sigma }{{E_{1} }} + \frac{\sigma }{{E_{2\infty } }} + \frac{{\sigma - \sigma_{s} }}{{\eta_{2i} }}t_{i} \hfill \\ \varepsilon_{j} = \frac{\sigma }{{E_{1} }} + \frac{\sigma }{{E_{2\infty } }} + \frac{{\sigma - \sigma_{s} }}{{\eta_{2i} }}t_{j} \hfill \\ \end{gathered} \right..$$

The above two equations are subtracted to obtain the difference in creep deformation of the rock at different times.

10 $$\varepsilon_{i} - \varepsilon_{j} = \frac{{\sigma - \sigma_{s} }}{{\eta_{2i} }}\left( {t_{i} - t_{j} } \right).$$

The viscosity coefficient *η*_2_ of the viscoplastic model is obtained in accordance with Eq. (10).

11 $$\eta_{2i} = \frac{{\sigma - \sigma_{s} }}{{\varepsilon_{i} - \varepsilon_{j} }}\left( {t_{i} - t_{j} } \right).$$

### Determination of viscoelastic modulus E_2_ and viscosity coefficient η_1_

When the applied stress is less than the long-term strength of the rock and the creep time *t* ≤ *t*_1_, the creep constitutive model of the rock is

12 $$\varepsilon = \frac{\sigma }{{E_{1} }} + \frac{\sigma }{{E_{2} }}\left[ {1 - \exp \left( { - \frac{{E_{2} }}{{\eta_{1} }}t} \right)} \right].$$

The primary creepcurve can take *n* time points *t*_*i*_, and a creep strain value *ε*_*i*_ corresponds to each time point *t*_*i*_. The elastic modulus *E*_1_ is unaffected by the creep time but degrades under different stresses. The creep equation at any time *t*_*i*_ can be expressed as

13 $$\varepsilon_{i} = \frac{\sigma }{{E_{1} }} + \frac{\sigma }{{E_{2i} }}\left[ {1 - \exp \left( { - \frac{{E_{2i} }}{{\eta_{1i} }}t_{i} } \right)} \right].$$

The first-order derivation of time *t* is given to Eq. (12), and the creep rate of the rock is obtained.

14 $$\varepsilon^{\prime} = \frac{\sigma }{{\eta_{1} }}\exp \left( { - \frac{{E_{2} }}{{\eta_{1} }}t} \right),$$

where *έ* is the creep rate.

The primary creep rate curve can take *n* time points *t*_*i*_, and a creep rate value *έ*_*i*_ corresponds to each time point *t*_*i*_.

15 $$\varepsilon^{\prime}_{i} = \frac{\sigma }{{\eta_{1i} }}\exp \left( { - \frac{{E_{2i} }}{{\eta_{1i} }}t_{i} } \right).$$

Equations (13) and (15) give the viscoelastic modulus *E*_2*i*_ and the viscosity coefficient* η*_1*i*_, respectively.

16 $$\left\{ \begin{gathered} \varepsilon_{i} - \frac{\sigma }{{E_{1} }} = - \frac{{\sigma t_{i} }}{{\eta_{1i} \ln \left( {\frac{{\varepsilon^{\prime}\eta_{1i} }}{\sigma }} \right)}}\left[ {1 - \frac{{\varepsilon^{\prime}_{i} \eta_{1i} }}{\sigma }} \right] \hfill \\ E_{2i} = - \ln \left( {\frac{{\varepsilon^{\prime}\eta_{1i} }}{\sigma }} \right)\frac{{\eta_{1i} }}{{t_{i} }} \hfill \\ \end{gathered} \right..$$

The *η*_1*i*_ term is expanded by Taylor series, and equation for calculating *η*_1*i*_ is

17 $$\frac{{\varepsilon_{i} E_{1} - \sigma }}{{t_{i} E_{1} }}\eta_{1i}^{3} + \frac{{\varepsilon_{i} E_{1} - \sigma }}{{t_{i} E_{1} }}\left[ {{\text{ln}}\left( {\frac{{\varepsilon^{\prime}_{i} }}{\sigma }} \right) - 1} \right]\eta_{1i}^{2} - \sigma \eta_{1i} + \varepsilon^{\prime}_{i} = 0.$$

Equation (17) can be transformed into a unary cubic polynomial, and the specific value of *η*_1*i*_ can be determined in accordance with a root finding equation. The specific value of *η*_1*i*_ is obtained and substituted into Eq. (16) to obtain the creep parameter *E*_2*i*_.

### Determination of the viscosity coefficient η_3_ of nonlinear Newtonian dashpot

When *σ* ≥ *σ*_*s*_ and *t* > *t*_2_, the creep constitutive model of rock is

18 $$\varepsilon = \frac{\sigma }{{E_{1} }} + \frac{\sigma }{{E_{2} }}\left[ {1 - \exp \left( { - \frac{{E_{2} }}{{\eta_{1} }}t} \right)} \right] + \frac{{\sigma - \sigma_{s} }}{{\eta_{2} }}t + \frac{\sigma }{{2\eta_{3} }}\left( {t - t_{2} } \right)^{2} .$$

The elastic moduli *E*_1*i*_ and *E*_2*i*_ and the viscous coefficients *η*_1*i*_ and *η*_2*i*_ corresponding to each time point *t*_*i*_ are determined. The accelerated creep curve can take *n* time points *t*_*i*_, and a creep strain value *ε*_*i*_ is found at each time point *t*_*i*_.

19 $$\varepsilon_{i} = \frac{\sigma }{{E_{1i} }} + \frac{\sigma }{{E_{2i} }}\left[ {1 - \exp \left( { - \frac{{E_{2i} }}{{\eta_{1i} }}t_{i} } \right)} \right] + \frac{{\sigma - \sigma_{s} }}{{\eta_{2i} }}t_{i} + \frac{\sigma }{{2\eta_{3i} }}\left( {t_{i} - t_{2} } \right)^{2} .$$

The viscous coefficient *η*_3*i*_ of the nonlinear Newtonian dashpot of the rock is determined in accordance with Eq. (19).

20 $$\eta_{3i} = \frac{{\sigma \left( {t_{i} - t_{2} } \right)^{2} }}{{2\left\{ {\varepsilon_{i} - \frac{\sigma }{{E_{1i} }} - \frac{\sigma }{{E_{2i} }}\left[ {1 - \exp \left( { - \frac{{E_{2i} }}{{\eta_{1i} }}t_{i} } \right)} \right] - \frac{{\sigma - \sigma_{s} }}{{\eta_{2i} }}t_{i} } \right\}}}.$$

Equations (7), (8), (9), (10), (11), (12), (13), (14), (15), (16), (17), (18), (19) and (20) are used to determine the parameters for the 1D viscoelastic-plastic creep model of rock.

## Promotion of 3D creep model parameter determination

### Establishment of 3D model

Under normal circumstances, the engineering rock mass in underground structures subjected to complex 3D stress. A triaxial compression rheological test is often conducted during the rock’s rheological experiment to better understand its stress state. This test provides information on the rocks’ mechanical properties^28^ and allows for the establishment of a suitable rheological constitutive model and the determination of rheological parameters for numerical analysis of the rock’s rheology. Therefore, a creep constitutive equation of rock under 3D stress must be established.

The 1D creep model of rock is transformed into a 3D model, and creep deformation can be transformed by analogy. The viscoplastic model involves the yield function *F* and the plastic potential function *Q*. The stress tensor *S*_*ij*_ cannot be used to replace the stress *σ* in the original 1D model, so it must be derived from the yield function. The yield function takes the following form during creep^29,30^.

21 $$F = \sqrt {J_{2} } - \sigma_{s} /\sqrt 3 ,$$

where *J*_2_ is the stress partial tensor second invariant.

The 3D equation of the viscoplastic model is expressed as

22 $$\varepsilon_{ij}^{vp} = \frac{1}{{\eta_{2} }}\left\langle {\frac{F}{{F_{0} }}} \right\rangle^{n} \frac{\partial Q}{{\partial \sigma_{ij} }}t,$$

where *F* is the yield function, *F*_0_ is the initial yield function value (generally taking *F*_0_ = 1), *n* is a constant (generally taking *n* = 1), and *Q* is the plastic potential function.

The abovementioned viscoelastic-plastic constitutive model based on statistical damage theory and 1D state is extended to 3D state as follows:

When *σ* < *σ*_*s*_,

23 $$\varepsilon_{11} = \frac{{\sigma {}_{1} - \sigma_{3} }}{{3G_{1} }} + \frac{{\sigma {}_{1} + 2\sigma_{3} }}{9K} + \frac{{\sigma {}_{1} - \sigma_{3} }}{{3G_{2} }}\left( {1 - \exp ( - \frac{{G_{2} }}{{\eta^{\prime}_{1} }})t} \right),$$

when *σ* ≥ *σ*_*s*_ and *t* < *t*_2_,

24 $$\varepsilon_{11} = \frac{{\sigma {}_{1} - \sigma_{3} }}{{3G_{1} }} + \frac{{\sigma {}_{1} + 2\sigma_{3} }}{9K} + \frac{{\sigma {}_{1} - \sigma_{3} }}{{3G_{2} }}\left( {1 - \exp ( - \frac{{G_{2} }}{{\eta^{\prime}_{1} }})t} \right) + \frac{{\sigma {}_{1} - \sigma_{3} - \sigma_{s} }}{{3\eta^{\prime}_{2} }}t,$$

when *σ* ≥ *σ*_*s*_ and *t* > *t*_2_,

25 $$\varepsilon_{11} = \frac{{\sigma {}_{1} - \sigma_{3} }}{{3G_{1} }} + \frac{{\sigma {}_{1} + 2\sigma_{3} }}{9K} + \frac{{\sigma {}_{1} - \sigma_{3} }}{{3G_{2} }}\left( {1 - \exp ( - \frac{{G_{2} }}{{\eta^{\prime}_{1} }})t} \right) + \frac{{\sigma {}_{1} - \sigma_{3} - \sigma_{s} }}{{3\eta^{\prime}_{2} }}t + \frac{{\sigma {}_{1} - \sigma_{3} }}{{6\eta^{\prime}_{3} }}\left( {t - t_{2} } \right)^{2} ,$$

where *G*_1_ and *G*_2_ are the shear moduli, *K* is the bulk modulus, and *η*'_1_, *η*'_2_, and *η*'_3_ are the viscous coefficients in a 3D state.

### Determination of 3D model parameters

When *t* → 0 +, the instantaneous strain of the rock in a 3D state is expressed as

26 $$\varepsilon_{11}^{e} = \frac{{\sigma {}_{1} - \sigma_{3} }}{{3G_{1} }} + \frac{{\sigma {}_{1} + 2\sigma_{3} }}{9K}.$$

The test results show that the instantaneous deformation includes elastic and plastic deformation. However, the instantaneous deformation is assumed to be elastic. The bulk modulus *K* at each stress state is determined by the instantaneous volumetric strain value of the test curve, which can be expressed as

27 $$K = \frac{{\sigma_{m} }}{{3\varepsilon_{m} }} = \frac{{\sigma_{m} }}{{3\varepsilon_{V} }} = \frac{{\sigma {}_{1} + 2\sigma_{3} }}{{9\varepsilon_{V} }},$$

where *σ*_m_ is the stress sphere tensor.

In accordance with the analogy method, the viscous coefficient *η*'_1_ and the viscoelastic modulus *G*_2*i*_ are obtained by combining Eqs. (16) and (17).

28 $$\varepsilon_{11}^{i} - \frac{{\sigma {}_{1} - \sigma_{3} }}{{3G_{1} }} - \frac{{\sigma {}_{1} + 2\sigma_{3} }}{9K} = \frac{{\left( {\sigma {}_{1} - \sigma_{3} } \right)t_{i} }}{{ - \left[ {{\text{ln}}\left( {\frac{{3\varepsilon^{\prime}_{i} }}{{\sigma {}_{1} - \sigma_{3} }}} \right) - {2}\eta_{1i} + 1} \right]3\eta_{1i} }}\left[ {1 - \frac{{3\eta^{\prime}_{1i} \varepsilon^{\prime}_{11i} }}{{\sigma {}_{1} - \sigma_{3} }}} \right].$$

29 $$G_{2i} = - \ln \left[ {\frac{{3\eta^{\prime}_{1i} \varepsilon^{\prime}_{11} }}{{\sigma {}_{1} - \sigma_{3} }}} \right]\frac{{\eta^{\prime}_{1i} }}{{t_{i} }}.$$

The viscosity coefficient *η*'_2*i*_ is obtained as

30 $$\eta^{\prime}_{2i} = \frac{{\sigma {}_{1} - \sigma_{3} - \sigma_{s} }}{{3\left( {\varepsilon_{11}^{i} - \varepsilon_{11}^{j} } \right)}}\left( {t_{i} - t_{j} } \right).$$

The viscous coefficient *η*'_3*i*_ corresponding to *ti* at any time point is determined as

31 $$\eta^{\prime}_{3i} = \frac{{\left( {\sigma {}_{1} - \sigma_{3} } \right)\left( {t_{i} - t_{2} } \right)^{2} }}{{6\left[ {\varepsilon_{11}^{i} - \frac{{\sigma {}_{1} - \sigma_{3} }}{{3G_{1} }} - \frac{{\sigma {}_{1} + 2\sigma_{3} }}{9K} - \frac{{\sigma {}_{1} - \sigma_{3} }}{{3G_{2i} }}\left( {1 - \exp ( - \frac{{G_{{2_{i} }} }}{{\eta^{\prime}_{1i} }})t_{i} } \right) - \frac{{\sigma {}_{1} - \sigma_{3} - \sigma_{s} }}{{3\eta^{\prime}_{2i} }}t_{i} } \right]}}.$$

## Indoor triaxial creep test of sandstone

### Test equipment and sandstone sample

**Figure 4:**
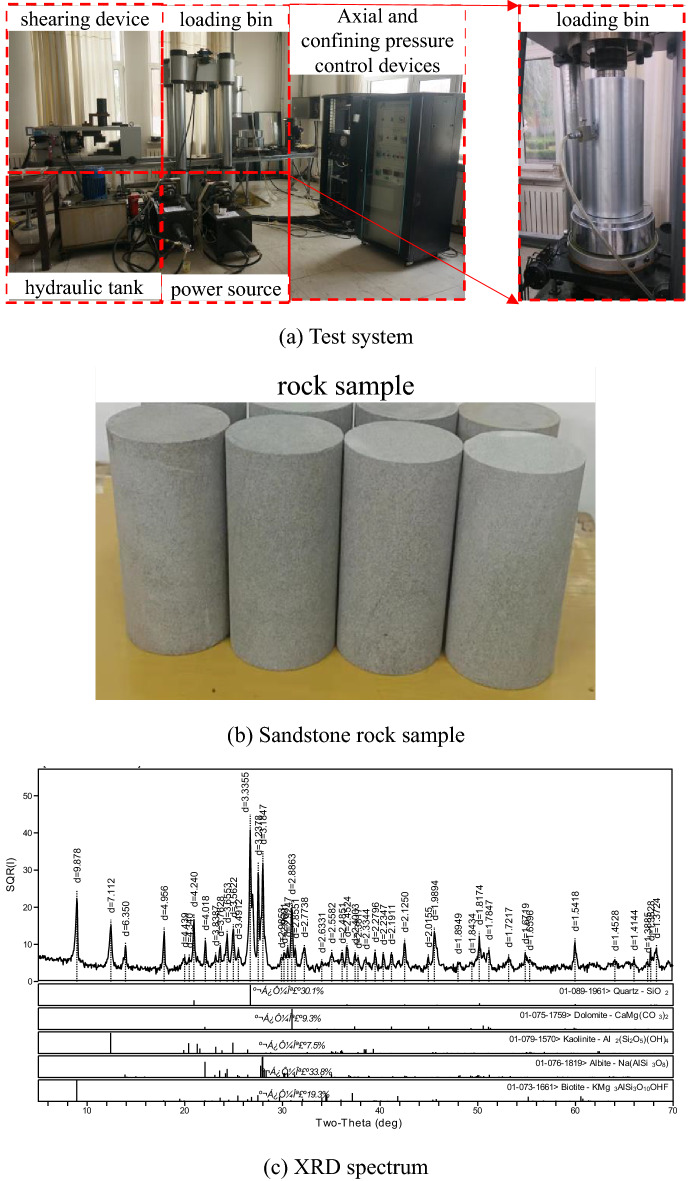
Rock mechanics test system and sandstone rock sample.

All the tests are conducted on a TAW-2000 triaxial testing machine (Fig. 4a). The test system consists of a loading part, a test part, and a control part. The sandstone samples used in the test are shown in Fig. 4b. The rock samples were collected from Hengda Coal Mine in Fuxin, Liaoning Province. The selected sandstone samples were buried at depths of 800 m to 850 m. The samples are dark gray in color, with a relatively uniform structure and a relatively hard texture. No visible microcracks and bedding are observed in the samples. Standard cylindrical specimens with a height of 100 mm and a diameter of 50 mm were processed in the laboratory. In accordance with the rock test rules of water conservancy and hydropower engineering, the density, water content, and natural water absorption of the sandstone were tested. The water content of the sandstone is determined to be 0.171% by the drying test. The natural water absorption of the sandstone is determined to be 2.349% by the free water absorption test. The density of the sandstone is determined to be 2.355 g/cm^3^ by the volumetric method. X-ray diffraction (XRD) analysis was conducted on the sandstone specimens in this test to better analyze the influence of the internal chemical composition and physical structure characteristics of the rock on its properties. The XRD results are shown in Fig. 4c.

As shown in Fig. 4c, the quartz content is 30.1%, the albite content is 33.8%, the dolomite content is 9.3%, the biotite content is 19.3%, and the kaolinite content is 7.5%.

### Conventional triaxial compression test

The specific test steps are as follows.

1. The triaxial conventional compression test of sandstone is conducted the conditions of 10 and 15 MPa.
2. Lateral stress is applied at a predetermined value with a loading rate of 500 N/s.
3. The test piece is subjected to hydrostatic stress, and the lateral stress is kept constant during the experiment.
4. The axial load is applied with a loading rate of 0.002 mm/s by using displacement control.
5. The testing machine automatically collects the test data and converts them corresponding strain and stress outputs for the data acquisition system.

**Figure 5:**
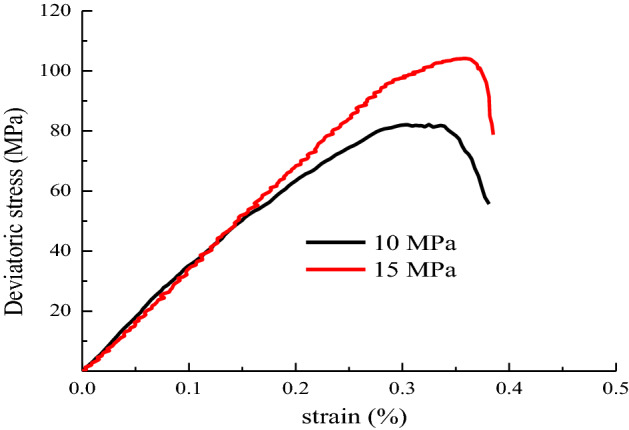
Deviatoric stress–strain relationship of conventional triaxial compression.

The sandstone stress–strain curve is drawn in accordance with the experimental data, as shown in Fig. 5.

As shown in Fig. 5, the peak strength of the sandstone and the elastic modulus increase with the increase in the confining stress. This condition results in the sandstone being more resistant to deformation and better able to withstand loads, making it less prone to damage.

### Creep test plan and analysis of creep results

The specific test steps are as follows.

1. The initial load for the sample with a confining pressure of 10 MPa is set to 40 MPa, and the initial load for the sample with a confining pressure of 15 MPa is set 50 MPa.
2. The load is increased in increments of 10 MPa, with a loading rate of 500 N/s.
3. The duration of each loading stage is determined in accordance with the strain deformation. When the axial deformation of the test piece measured within 2 h is less than 0.001 mm, the deformation is considered to be relatively stable.
4. The next loading stage is applied, and the process is repeated until the sample becomes unstable.

**Figure 6:**
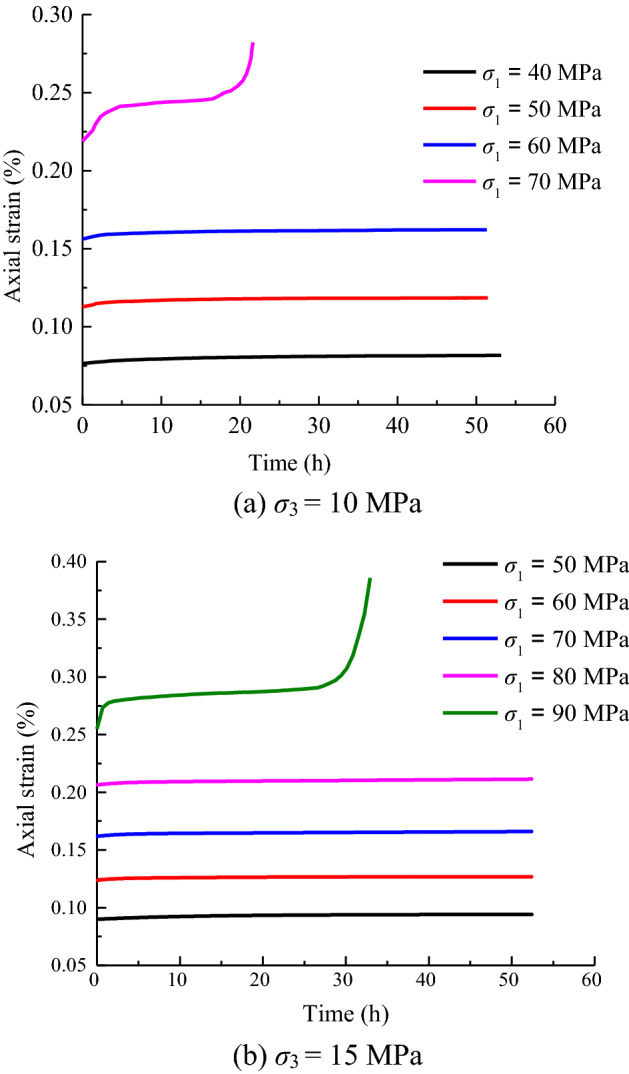
Creep of axial under different axial stress states.

The experimental data are superimposed by using Chen’s superposition principle, and the corrected axial deformation duration curve is obtained, as shown in Fig. 6.

As shown in Fig. 6, the axial strain of the test piece is generated instantaneously at the axial stress level. The magnitude of the instantaneous strain is related to the stress level, with higher stress levels resulting in larger instantaneous strain values. However, the ratio of instantaneous strain to total strain increases first and then decreases. This behavior is probably caused by the extrusion closure of internal voids in the initial loading stage and subsequent crack propagation in later stages.

The creep rate is the slope of the creep test curve at each data point. However, due to the non-uniformity, anisotropy and micro-defects of the sample during the experiment, the test data oscillated. The determination of creep rate from the original test data is bound to cause the development trend of creep rate to fluctuate greatly. It is not conducive to analyze the creep rate and difficult to reflect the creep deformation law. The axial-radial and volume creep curves under different levels of stress under various confining pressure conditions are nonlinearly fitted by Origin software. It can fully reflect the creep development trend. Then the derivative operation is performed on each empirical equation. The more regular creep rate development trend can also be obtained. It also avoids data oscillation and beating. The creep rate is treated by the method in Reference^31,32^.

**Figure 7:**
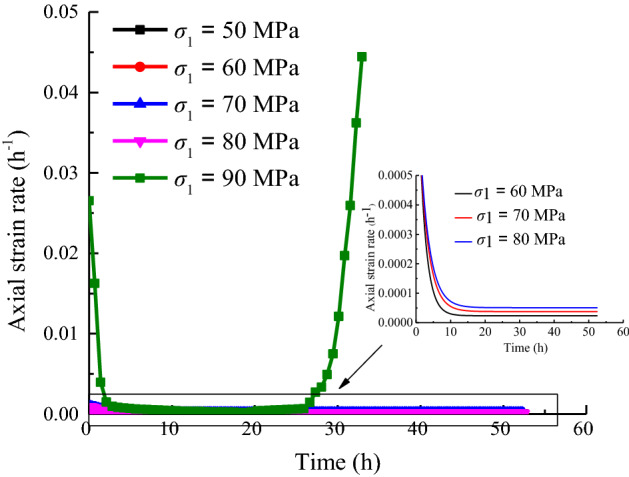
Creep strain rate when *σ*_3_ = 15 MPa.

In accordance with the basic concept of creep, the creep speed can be defined as the slope of the creep test curve at each data point. The test data may exhibit oscillations due to the nonuniformity, anisotropy, and microdefects of the sample during the experiment. A creep rate curve with a confining pressure of 15 MPa is shown in Fig. 7.

As shown in Fig. 7, the creep rate under each stage of stress decreases initially and gradually stabilizes over time. However, the creep rate begins to increase with time when the rock enters the accelerated creep phase.

**Figure 8:**
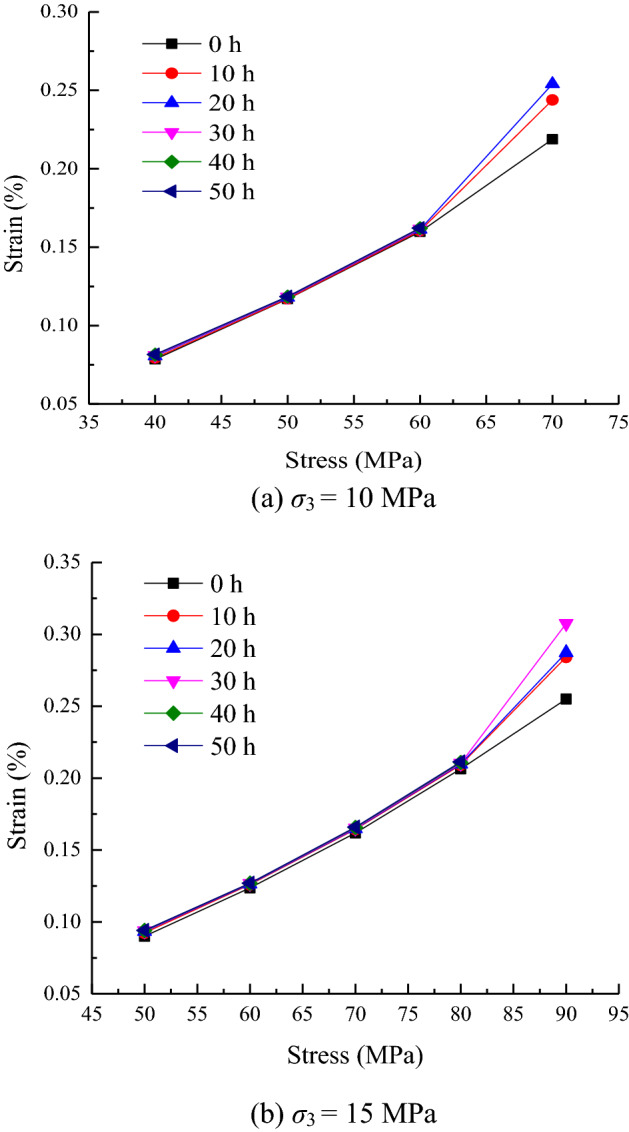
Isochronous stress–strain curves.

An isochronous stress–strain curve represents the relationship between stress and strain at the same time and is depicted as a cluster of divergent polyline segments. The long-term strength of the rock can be determined by the stress value corresponding to the inflection point of the isochronous stress–strain curve. This curve is drawn in accordance with the creep-duration curve, as shown in Fig. 8.

As shown in Fig. 8, the isochronous stress–strain curve is a cluster of divergent broken line segments. The strain value of the isochronous stress–strain curve increases with the increase in stress. Until the stress value reaches 80 MPa, the curve appears as a coincident line, and the stress–strain relationship can be considered linear. Beyond 80 MPa, the curve becomes divergent, and the degree of divergence increases continuously with the increase in stress over time. In this case, the stress–strain relationship can be considered nonlinear. The stress value of 80 MPa marks the transition from linear deformation to nonlinear deformation. Therefore, the stress value corresponding to this point can be regarded as the long-term strength value of the rock.

## Analysis of the variation law of creep parameters

### Creep parameter determination

In practical engineering, the physical and mechanical parameters of rock under complex geological conditions obviously changes with stress and time. Parameters, such as the modulus of elasticity, strength, and viscosity, decrease over time. Therefore, the creep parameter is expressed as a functional expression of stress versus time better reflects the essential properties of rock materials. Under constant stress, the creep parameters are considered to be time-dependent. The shear modulus *G*_1_ is constant and not affected by time under constant stress. The bulk modulus *K* can be obtained from the test data and Eq. (27). For other creep parameters under various stress conditions, the values at *t* = 0, 5, 10, 15, 20 and 25 h under the previous load and under the last stage load are determined by combining the test curves and the duration of the load. The creep parameters of rock under different stress levels and at different times are calculated by combining the results of triaxial indoor creep test and using the method for determining the parameters of the 3D viscoelastic-plastic creep model of rock from Eqs. (28), (29), (30) and (31).

### Analysis of the variation law of shear modulus G_1_

The instantaneous shear modulus has the following relationship with the instantaneous stress tensor *S*_*ij*_ and the instantaneous strain tensor *e*_*ij*_.

32 $$S_{ij} = 2G_{1} e_{ij} .$$

**Figure 9:**
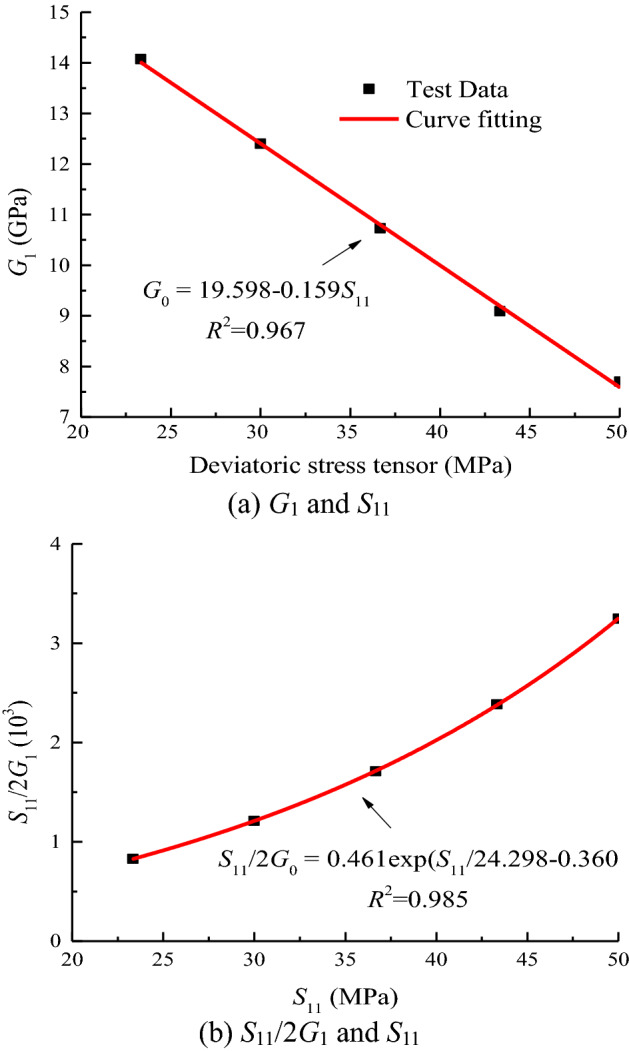
Data fitting of *G*_1_ when *σ*_3_ = 15 MPa.

The relationship between these variables is plotted in Fig. 9. Equation (33) represents the linear relationship between the instantaneous shear modulus of the rock and the stress tensor.

33 $$G_{1} = 10^{3} \times \,\left( {a_{1} - b_{1} S_{11} } \right),$$

where *a*_1_and *b*_1_ are fitting parameters.

As shown in Fig. 9, the shear modulus *G*_1_ of the rock decreases with the increase in the applied bias stress tensor *S*_*ij*_. This condition embodies the nonlinear damage effect of rock loading through progressive loading. With the increase in the bias stress tensor *S*_*ij*_, the strain tensor *e*_*ij*_ = *S*_*ij*_/2*G*_1_ gradually increases, indicating that the greater the stress tensor, the greater the change in rock shape.

### Analysis of the variation law of bulk modulus K

**Figure 10:**
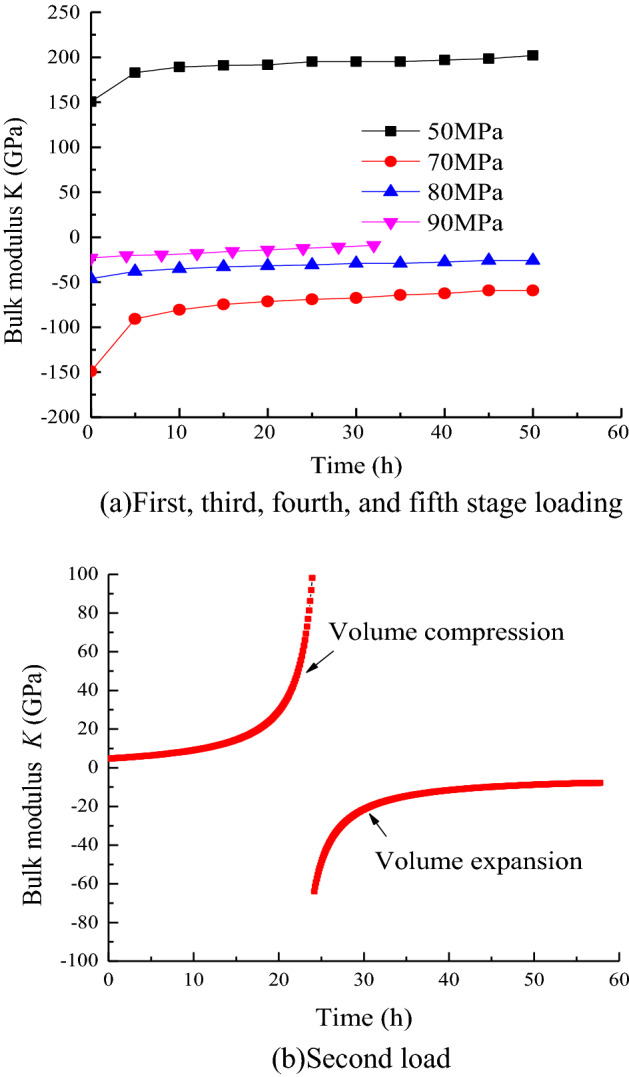
Duration of creep and bulk modulus.

In accordance with Table 4, the bulk modulus of the rock has a negative value between the stress level of 60 MPa and the creep time of 5 h to 10 h. The volume modulus *K* at each moment under the action of the stress level of 60 MPa is obtained by using Eq. (27). The relationship between the bulk modulus *K* and time under the load of each stage is shown in Fig. 10.

As shown in Fig. 10, the change in the bulk modulus *K* value with time reflects the shrinkage and expansion of the rock volume from the side. When the axial stress is 50 MPa, *K* is positive and increased with time. The rate of increase gradually decreases and eventually stabilizes, indicating that the volume of the sample is in a compressed state relative to its original size, and the volume deformation is continuously reducing. Under the axial stress of 60 MPa, the volume strain is approximately zero and slowly decreases, suggesting a gradual transition from compression to expansion in the volume deformation. At the creep time of 24.17 h, the bulk modulus changes from positive infinity to negative infinity, reflecting a transition from a positive volumetric strain to a negative one, and a change from compression to expansion in the volume change. When *t* is less than 24.17 h, the bulk modulus *K* is positive and gradually increases from 113.56 GPa to positive infinity over time. When *t* is greater than 24.17 h, the bulk modulus *K* is negative and gradually increases from negative infinity to − 63.89 GPa over time. The bulk modulus *K* is negative under axial stresses of 70, 80 and 90 MPa. With the increase in the stress level, the absolute value decreases continuously. The rate of increase in *K* value increases with time, indicating that the sample is in the stage of expansion, and the expansion effect becomes increasingly obvious with the improvement of the stress level and the development of time.

In accordance with the distribution characteristics of *K*/*σ*_m_ with time, the variation of creep parameters under a stress level of 60 MPa is fitted in accordance with Eq. (34), and the bulk modulus under other loads is fitted in accordance with Eq. (35).

**Figure 11:**
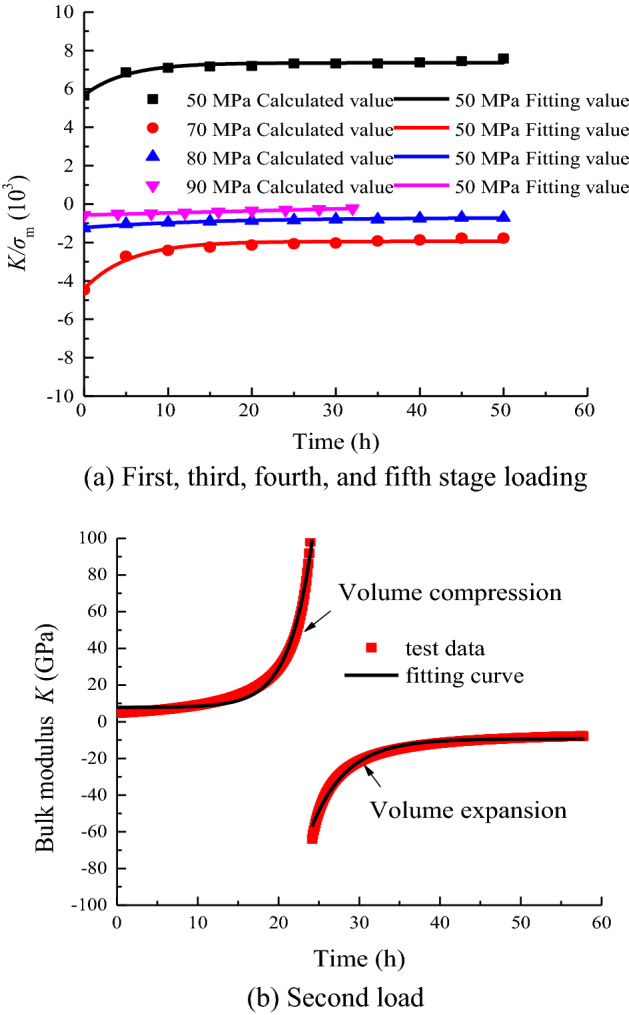
Bulk modulus.

The variation law of the volume modulus *K* with time *t* is shown in Fig. 11. The fitting parameters for each load condition are shown in Table 1.

34 $$\frac{K}{{\sigma_{{\text{m}}} }} = a_{2} {\text{exp}}\left( { - t/b_{2} } \right) + c_{2} ,$$

where *a*_2_, *b*_2_, *c*_2_, *a*_3_, *b*_3_, and *c*_3_ are fitting parameters.

**Table 1:** Fitting parameters under loads.

Stress level/MPa		*a*_3_ (*a*_2_)	*b*_3_ (*b*_2_)	*c*_3_ (*b*_2_)	*R*^2^
50		− 1.686	4.946	7.357	0.953
60	Positive value	0.019	− 2.859	7.854	0.982
	Negative value	− 12,357.000	4.346	− 9.298	0.985
70		− 2.479	5.359	− 1.940	0.965
80		− 0.51704	15.914	− 0.702	0.965
90		− 11,688.433	1.090*10^6^	11,687.865	0.993

### Analysis of the variation law of viscoelastic shear modulus G_2_

**Figure 12:**
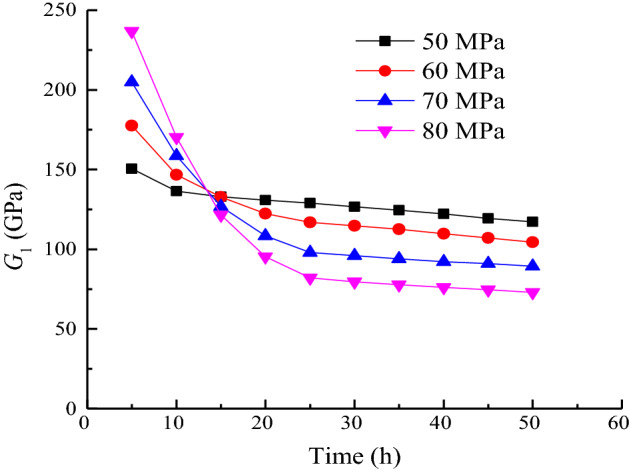
Duration of *G*_1_ when *σ*_3_ = 15 MPa.

The relationship between the viscoelastic shear modulus *G*_2_ and the time *t* under each stage of loading is shown in Fig. 12.

As shown in Fig. 12, *G*_2_, which controls the limit viscoelastic deformation of the rock sample, gradually decreases with time. The absolute value of the slope of the curve gradually decreases, indicating that the viscoelastic deformation gradually increases with time and tends to a stable value. At the initial stage of loading, the viscoelastic shear modulus *G*_2_ increases with increasing stress level. In the later stage of loading, the higher the stress level, the smaller the value of *G*_2_. The higher the stress level, the larger the magnitude of the change in *G*_2_. In accordance with the distribution characteristics of *S*_11_/*G*_1_ over time, the creep parameters under different stress levels are fitted by Eq. (36).

**Figure 13:**
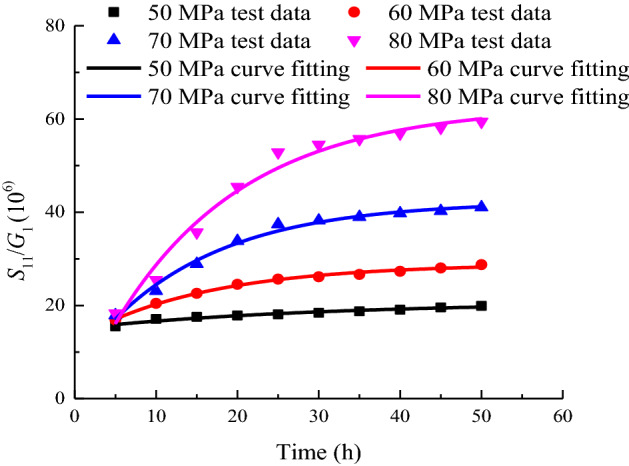
Fitting of *S*_11_/*G*_2_ contrast experiment curves when *σ*_3_ = 15 MPa.

The variation law is shown in Fig. 13. The fitting parameters under each load are shown in Table 2.

35 $$\frac{{S_{11} }}{{G_{2} }} = a_{4} {\text{exp}}\left( { - t/b_{4} } \right) + c_{4} ),$$

where *a*_4_, *b*_4_, and *c*_4_ are fitting parameters.

**Table 2:** Calculation result of *S*_11_/*G*_1_.

Stress level/MPa	50	60	70	80
*a*_4_	− 5.743	− 16.211	− 35.647	− 63.886
*b*_4_	30.442	16.061	14.447	15.958
*c*_4_	20.770	28.940	42.295	62.834
*R*^2^	0.943	0.992	0.987	0.977

The value of *S*_11_/*G*_2_ represents the trend of the axial viscoelastic strain of the sample. As shown in Fig. 13, the longer the time and the higher the stress level, the greater the final viscoelastic strain.

### Analysis of the variation law of viscous coefficient η'_1_

**Figure 14:**
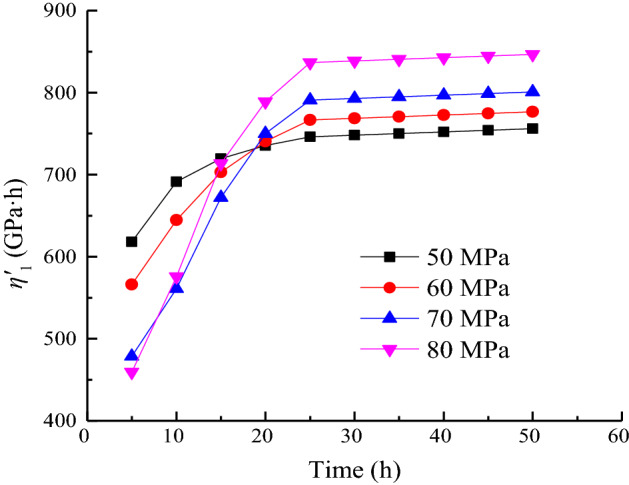
Duration of *η*_1_ when *σ*_3_ = 15 MPa.

The relationship between the shear viscoelastic coefficient *η*'_1_ and time at different loads is shown in Fig. 14.

As shown in Fig. 14, the shear viscoplastic coefficient *η*'_1_ decreases with time under the same load, indicating that the creep rate gradually decreases with time and tends to a stable value. The creep of the sample enters the steady-state creep stage. The higher the stress level, the larger the magnitude of the change in *η*'_1_.

**Figure 15:**
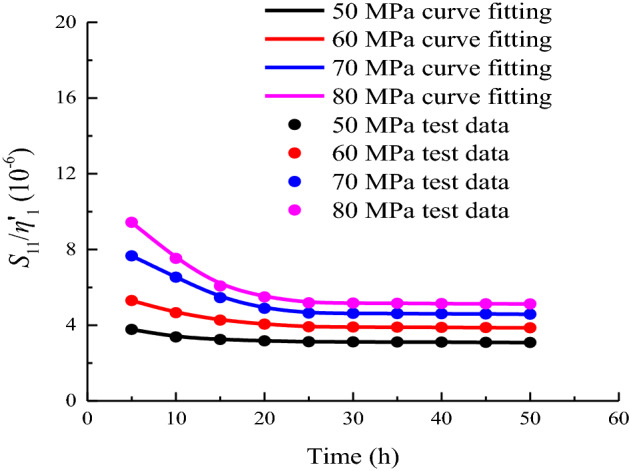
Data fitting of *S*_11_/*η*^*’*^_1_ when *σ*_3_ = 15 MPa.

In accordance with the distribution characteristics of *S*_11_/*η*'_1_ with time, the creep parameters under different stress levels are fitted by Eq. (37). The variation law is shown in Fig. 15. The fitting parameters under each load are shown in Table 3.

36 $$\frac{{S_{11} }}{{\eta^{\prime}_{1} }} = a_{5} {\text{exp}}\left( { - t/b_{5} } \right) + c_{5} ,$$

where *a*_5_, *b*_5_, and *c*_5_ are fitting parameters.

**Table 3:** Calculation result of *S*_11_/*η*_1_.

Stress level/MPa	50 MPa	60 MPa	70 MPa	80 MPa
*a*_5_	1.518	2.766	5.928	8.906
*b*_5_	6.092	7.841	8.330	7.224
*c*_5_	3.101	3.850	4.499	5.050
*R*^2^	0.996	0.997	0.983	0.991

### Analysis of the variation law of viscous coefficient η'_2_

**Figure 16:**
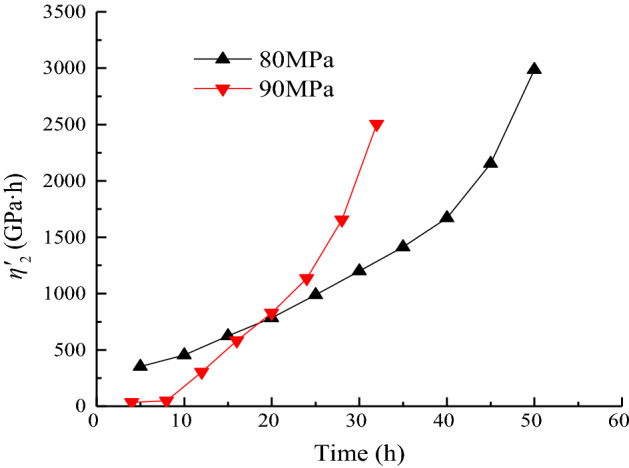
Duration of *η*^*’*^_2_ when *σ*_3_ = 15 MPa.

The plastic element only operates when *σ*_1_ − *σ*_3_ ≥ *σ*_s_. The relationship between the shear viscoplastic coefficient *η*'_2_ and time *t* at each moment under different loads is shown in Fig. 16.

As shown in Fig. 16, *η*'_2_ increases gradually with time under the same load, and the rate of viscoplastic creep decreases with time. When the axial stress is 80 MPa, the shear viscoplastic coefficient *η*^*’*^_2_ is higher than when under the action of 90 MPa. It shows that the rate of the strain-steady-state creep stage of the rock under action of 80 MPa is much less than 90 MPa. At the initial stage of loading, the shear viscoplastic coefficient *η*^*’*^_2_ increases with the increase in stress level. At the end of loading, *η*^*’*^_2_ decreases with increasing stress level. This condition is due to the greater internal reorganization of the rock’s viscosity as the stress level increases in the primary creepstage. As time progresses into a steady-state creep phase, the internal structure damage is more severe, and the load causes the viscosity to decrease.

**Figure 17:**
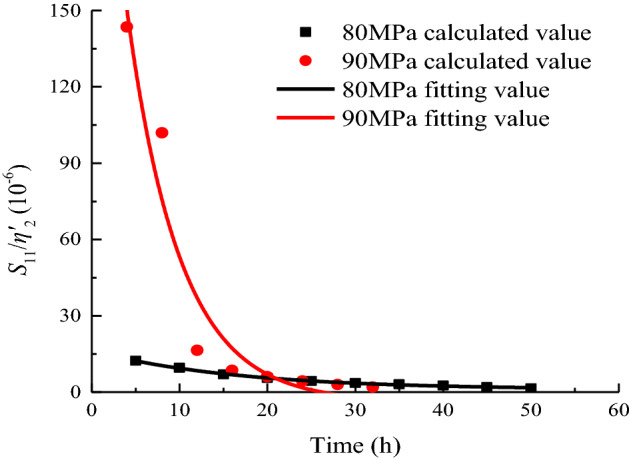
Data fitting of *S*_11_/*η*^*’*^_2_ when *σ*_3_ = 15 MPa.

In accordance with the distribution characteristics of *S*_11_/*η*^*’*^_2_ with time, the creep parameters under the stress levels of 80 and 90 MPa are fitted by Eq. (37). The variation law is shown in Fig. 17. The fitting parameters under each load are shown in Table 4.

37 $$\frac{{S_{11} }}{{\eta^{\prime}_{2} }} = a_{6} {\text{exp}}\left( { - t/b_{6} } \right) + c_{6} ,$$

where *a*_6_, *b*_6_, and *c*_6_ are fitting parameters.

**Table 4:** Calculation result of *S*_11_/*η*^*’*^_2_.

Stress level/MPa	80	90
*a*_6_	15.244	300.045
*b*_6_	16.752	6.021
*c*_6_	0.980	− 3.829
*R*^2^	0.997	0.915

The value of *S*_11_/*η*^*’*^_2_ mainly controls the rate of axially strained viscoplastic deformation of the specimen. As shown in Fig. 17, the higher the stress level, the greater the rate of viscoplastic deformation creep. The rate of viscoplastic deformation of the rock samples decreases with time and gradually stabilizes, and the rock creep enters a steady-state creep stage.

### Analysis of the variation law of viscous coefficient *η*'_3_

**Figure 18:**
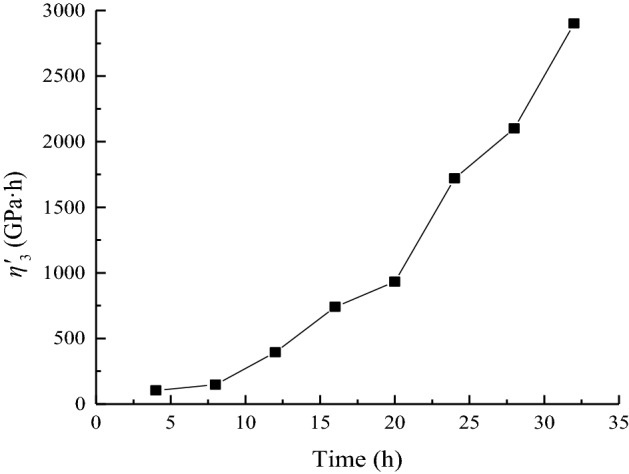
Duration of *η*^*’*^_3_ when *σ*_3_ = 15 MPa.

Only when *t* ≥ *t*_2_, the nonlinear Newtonian dashpot starts to generate creep deformation. Therefore, the acceleration creep stage appears only when the stress level is 90 MPa. The relationship between the shear viscoplastic coefficient *η*'_3_ at each moment and the time *t* is plotted in Fig. 18.

**Figure 19:**
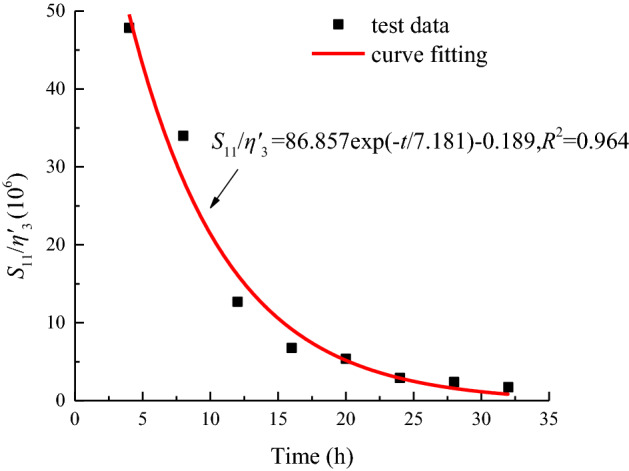
Data fitting of *S*_11_/*η*^*’*^_3_ when *σ*_3_ = 15 MPa.

In accordance with the distribution characteristics of *S*_11_/*η*^*’*^_3_ with time, the creep parameters under the stress levels of 90 MPa is fitted by Eq. (39). The variation law is shown in Fig. 19.

38 $$\frac{{S_{11} }}{{\eta^{\prime}_{3} }} = a_{7} {\text{exp}}\left( { - t/b_{7} } \right) + c_{7} ,$$

where *a*_7_, *b*_7,_ and *c*_7_ are fitting parameters.

The viscous coefficient *η*^*’*^_3_ mainly affects the accelerated creep stage. As shown in Fig. 19, *S*_11_/*η*^*’*^_3_ gradually decreases with time.

## Determination and verification of creep model

### Determination of creep model under the influence of stress state and time

In this research, the creep parameters of the transient creep, primary creepphase, steady-state creep phase, and accelerated creep phase are analyzed with the stress level and time. A mathematical model is derived to describe the relationship between the creep parameters, stress, and time. This creep parameter model is substituted into Eqs. (23), (24) and (25) to obtain a creep 3D model that considers the stress state and time.

When *σ* < *σ*_*s*_,

39 $$\varepsilon_{11} = \frac{{\sigma {}_{1} - \sigma_{3} }}{{3G_{1} \left( {S_{ij} } \right)}} + \frac{{\sigma {}_{1} + 2\sigma_{3} }}{{9K\left( {\sigma_{{\text{m}}} ,t} \right)}} + \frac{{\sigma {}_{1} - \sigma_{3} }}{{3G_{2} \left( {S_{ij} ,t} \right)}}\left( {1 - \exp \left( - \frac{{G_{2} \left( {S_{ij} ,t} \right)}}{{\eta^{\prime}\left( {S_{ij} ,t} \right)_{1} }}\right)t} \right),$$

when *σ* ≥ *σ*_*s*_ and *t* < *t*_2_,

40 $$\varepsilon_{11} = \frac{{\sigma {}_{1} - \sigma_{3} }}{{3G_{1} \left( {S_{ij} } \right)}} + \frac{{\sigma {}_{1} + 2\sigma_{3} }}{{9K\left( {\sigma_{{\text{m}}} ,t} \right)}} + \frac{{\sigma {}_{1} - \sigma_{3} }}{{3G_{2} \left( {S_{ij} ,t} \right)}}\left( {1 - \exp\left ( - \frac{{G_{2} \left( {S_{ij} ,t} \right)}}{{\eta^{\prime}_{1} \left( {S_{ij} ,t} \right)}}\right)t} \right) + \frac{{\sigma {}_{1} - \sigma_{3} - \sigma_{s} }}{{3\eta^{\prime}_{2} \left( {S_{ij} ,t} \right)}}t,$$

when *σ* ≥ *σ*_*s*_ and *t* > *t*_2_,

41 $$\varepsilon_{11} = \frac{{\sigma {}_{1} - \sigma_{3} }}{{3G_{1} \left( {S_{ij} } \right)}} + \frac{{\sigma {}_{1} + 2\sigma_{3} }}{{9K\left( {\sigma_{{\text{m}}} ,t} \right)}} + \frac{{\sigma {}_{1} - \sigma_{3} }}{{3G_{2} \left( {S_{ij} ,t} \right)}}\left( {1 - \exp \left( - \frac{{G_{2} \left( {S_{ij} ,t} \right)}}{{\eta^{\prime}\left( {S_{ij} ,t} \right)_{1} }}\right)t} \right) + \frac{{\sigma {}_{1} - \sigma_{3} - \sigma_{s} }}{{3\eta^{\prime}_{2} \left( {S_{ij} ,t} \right)}}t + \frac{{\sigma {}_{1} - \sigma_{3} }}{{6\eta^{\prime}_{3} \left( {S_{ij} ,t} \right)}}\left( {t - t_{2} } \right)^{2} .$$

### Verification of creep model

**Figure 20:**
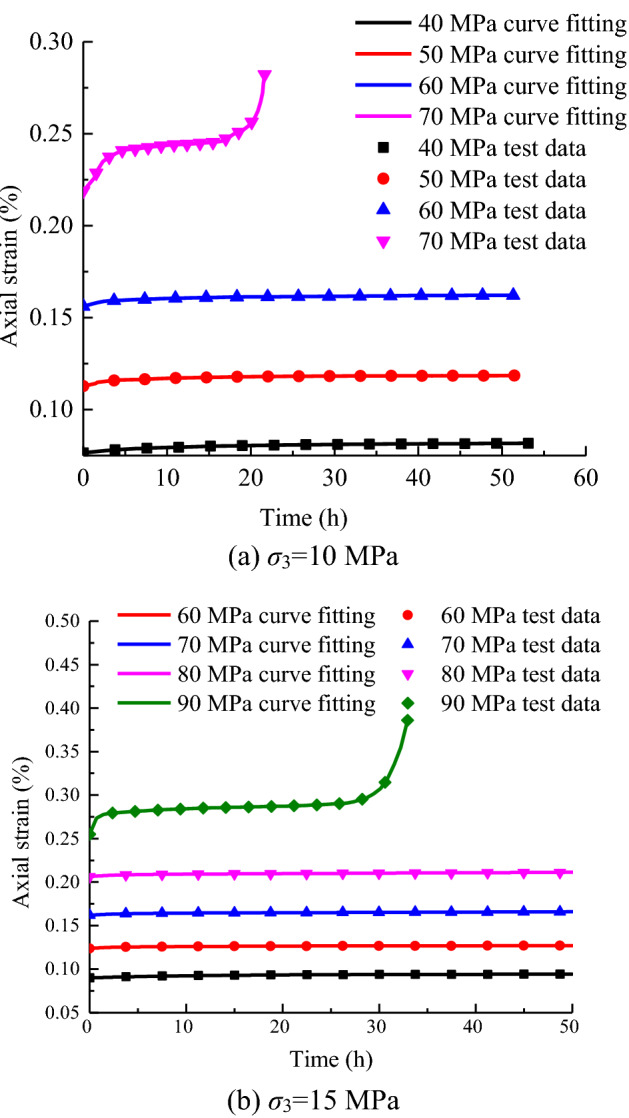
Experimental results compared with the calculated values.

The curves of different stress levels and creep models at various times are plotted in Eqs. (39), (40) and (41) and compared with the experimental data, as shown in Fig. 20.

As shown in Fig. 20, the model curve conforms to the variation curve of the creep test at confining pressures of 10 and 15 MPa. The high consistency between the model curve and the test curve indicates that substituting the method of calculating the creep parameters into the creep model is suitable and feasible to reflect the deformation process of rock creep. The model accurately reflects the creep characteristics of the primary and steady-state creep stages, and overcomes the shortcomings of traditional creep models, which are difficult to describe the accelerated creep. The model has a good predictive analysis of triaxial creep test data.

## Verification and analysis of theoretical models for other types of rocks

**Figure 21:**
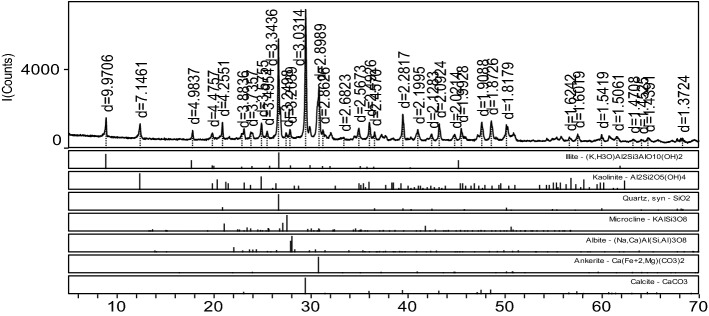
XRD pattern of the rock sample.

The above analysis and verification results are based on sandstone. This study selects the surrounding rock of the No. 2 tunnel of Dali No. 1 section as the test object to verify the correctness and rationality of the proposed theoretical model. The rock is preliminarily determined as chlorite schist. The rock samples mainly contain quartz, kaolinite, illite, dolomite, and calcite. The XRD pattern of the rock sample is shown in Fig. 21.

**Figure 22:**
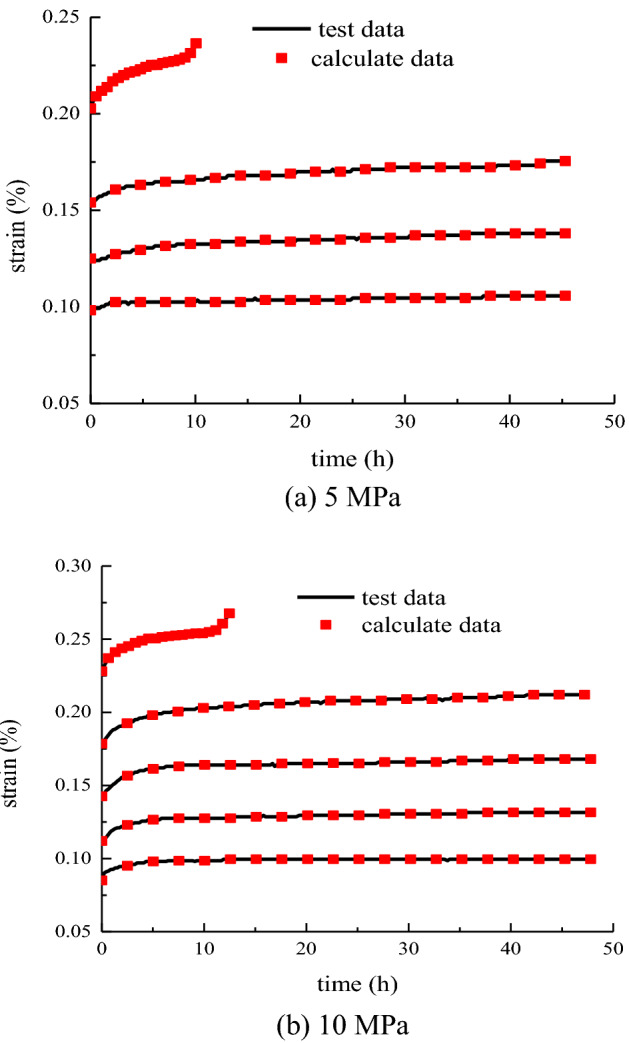
Comparison of creep model and test curve.

In accordance with the above steps and the creep test data of the rock, the model parameters under different times and stresses are calculated. The model parameters are substituted into Eqs. (39), (40) and (41) to obtain the rock model curve. The model curve is drawn and compared with the experimental curve, as shown in Fig. 22.

As shown in Fig. 22, the creep model curve of the rock is highly fitted with the test curve, describing the whole process of decay creep, constant creep, and accelerated creep. This condition makes up for the shortcomings of traditional models in describing accelerated creep. The method of determining creep parameters and the new creep model proposed in this study can be applied to different types of rocks.

## Conclusions

A method for determining creep parameters by creep curves is proposed. Various creep parameter models under different stresses and different times are established by assuming certain conditions. A creep model that can correspond to each creep stage is established. The model of creep parameters with respect to time and stress is substituted into the traditional creep model to create a time-dependent creep model that considers the stress state. The model curve conforms to the variation curve of the creep test at confining pressures of 10 and 15 MPa. The high consistency of the model curve and the test curve indicates that substituting the method of calculating the creep parameters into the creep model is suitable and feasible to reflect the deformation process of rock creep. Overall, the model has a good predictive analysis of triaxial creep test data.

The relationship between creep parameters and stress levels is unclear. The change in creep parameters with time reflects the deterioration of the rock structure to some extent. The shear modulus *G*_1_ of the rock decreases with the increase in the applied bias stress tensor *S*_*ij*_. Under the axial stress of 60 MPa, the relationship between bulk modulus and time is divided into two parts. The bulk modulus is positive, and its relationship with time shows a trend of steady increase first and then rapid increase. The bulk modulus is negative, and its relationship with time shows a trend of increasing first and then stabilizing. The shear viscoplastic coefficient *η*'_1_ decreases with time under the same load. The value of *S*_11_/*G*_2_ represents the development trend of the axial viscoelastic strain of the sample. The longer the time and the higher the stress level, the greater the final viscoelastic strain. The *η*'_2_ gradually increases with time under the same load. The slope of the curve gradually increases. The value of *S*_11_/*η*^*’*^_2_ mainly controls the rate of axially strained viscoplastic deformation of the specimen. The viscous coefficient *η*^*’*^_3_ mainly affects the accelerated creep stage, and the *S*_11_/*η*^*’*^_3_ gradually decreases with time.

## Data Availability

The datasets used in this study are available upon reasonable request from the corresponding author.
